# Linkage disequilibrium in Angus, Charolais, and Crossbred beef cattle

**DOI:** 10.3389/fgene.2012.00152

**Published:** 2012-08-14

**Authors:** Duc Lu, Mehdi Sargolzaei, Matthew Kelly, Changxi Li, Gordon Vander Voort, Zhiquan Wang, Graham Plastow, Stephen Moore, Stephen P. Miller

**Affiliations:** ^1^Centre for Genetic Improvement of Livestock, Department of Animal and Poultry Science, University of GuelphGuelph, ON, Canada; ^2^Lacombe Research Centre, Agriculture and Agri-Food CanadaLacombe, AB, Canada; ^3^Department of Agricultural, Food and Nutritional Science, University of AlbertaEdmonton, AB, Canada; ^4^Centre for Animal Science, Queensland Alliance for Agriculture and Food Innovation, University of QueenslandSt. Lucia, QLD, Australia

**Keywords:** beef cattle, linkage disequilibrium

## Abstract

Linkage disequilibrium (LD) and the persistence of its phase across populations are important for genomic selection as well as fine scale mapping of quantitative trait loci (QTL). However, knowledge of LD in beef cattle, as well as the persistence of LD phase between crossbreds (C) and purebreds, is limited. The objective of this study was to understand the patterns of LD in Angus (AN), Charolais (CH), and C beef cattle based on 31,073, 32,088, and 33,286 SNP in each population, respectively. Amount of LD decreased rapidly from 0.29 to 0.23 to 0.19 in AN, 0.22 to 0.16 to 0.12 in CH, 0.21 to 0.15 to 0.11 in C, when the distance range between markers changed from 0–30 kb to 30–70 kb and then to 70–100 kb, respectively. Breeds and chromosomes had significant effects (*P* < 0.001) on LD decay. There was significant interaction between breeds and chromosomes (*P* < 0.001). Correlations of LD phase were high between C and AN (0.84), C and CH (0.81), as well as between AN and CH (0.77) for distances less than or equal to 70 kb. These dropped when the distance increased. Estimated effective population sizes for AN and CH were 207 and 285, respectively, for 10 generations ago. Given a useful LD of at least 0.3 between pairs of SNPs, the LD phase between any pair of the three breed groups was highly persistent. The current SNP density would allow the capture of approximately 49% of useful LD between SNP and marker QTL in AN, and 38% in CH. A higher density SNP panel or redesign of the current panel is needed to achieve more of useful LD for the purpose of genomic selection beef cattle.

## Introduction

Linkage disequilibrium (LD) refers to non-random association of alleles at two or more loci, and is important in fine scale mapping of quantitative trait loci (QTL) (Meuwissen and Goddard, 2000). Exploitation of LD results in the improvement of genetic gains in marker-assisted selection schemes (Schulman and Dentine, 2005). Understanding LD aids in the optimal design of marker panels that make the most use of the available LD in the population being studied or selected. With genomic selection the values of markers discovered in a reference population may be used as predictors in other populations.

Selection to improve livestock performance has been practiced hand in hand with controlling inbreeding rates. At the individual level, inbreeding is the result of deliberate mating of related individuals. At the population level, random genetic drift causes the division of a population into subpopulations with a smaller number of parents, thus results in inbreeding. At the molecular level, random drift affects allele frequencies, leads to loss of neutral genetic variation, and fixation of deleterious or favorable alleles. An approach that helps predict these losses is effective population size (*N*_*e*_), which is defined by Wright (1938) as the number of breeding individuals in an idealized population that would show the same amount of dispersion of allele frequencies under random genetic drift or the same amount of inbreeding as the population under consideration. Thus, estimate of *N*_*e*_ should be considered when making decisions concerning selection pressure. However, reliable estimates of *N*_*e*_ from demographic parameters are difficult to achieve (Frankham, 1995), thus predicting *N*_*e*_ from LD between loci is an option.

Genomic selection uses marker effects estimated in a reference population to predict breeding values (BV) of selection candidates based on their marker genotypes (Meuwissen et al., 2001). In beef cattle the application of genomic selection is still developing. Unlike the situation in dairy cattle the benefit of genomic selection in beef cattle will come in part from traits that are not part of routine industry performance recording programs. Traits that are important, but expensive and difficult to measure such as feed efficiency are an important component of efforts in beef cattle. These data are largely based around research populations including the one used in this study, which is crossbred. An example of this is the work of Snelling et al. (2010), in which the effect of SNP on growth rate was estimated in crossbred beef cattle. In this case, marker effects are estimated in crossbred populations, and ideally these estimates could be used to predict BV of purebred animals and animals of other crossbred populations for selection purposes. The accuracy of the BV predicted this way depends on the persistence of LD phase between the crossbred and the purebred populations (Dekkers and Hospital, 2002; Goddard et al., 2006).

There has been extensive research about LD in purebred cattle with Holstein and Angus (AN) being the main focus (Odani et al., 2006; McKay et al., 2007; de Roos et al., 2008; Khatkar et al., 2008; Marques et al., 2008; Prasad et al., 2008; Sargolzaei et al., 2008; Bohmanova et al., 2010). Kim and Kirkpatrick (2009) reported LD of greater 0.80 over genomic regions of approximately 50 kb using 7119 SNP on 200 North American Holstein cattle. Meanwhile Qanbari et al. (2010) reported an average LD of 0.30 over pairwise distances of less than 25 kb, using 40,854 SNP on 810 German Holstein-Friesian cattle. Apparently SNP density and sample size had played their role in the outputs of those two studies. For beef cattle, studies on AN and other beef cattle breeds were conducted with less dense marker panels, for instance 2670 makers (McKay et al., 2007), 500 SNP (Marques et al., 2008), 246 microsatellite markers (Odani et al., 2006), and on small groups of animals, for instance 90 AN and 40 Charolais (CH) (McKay et al., 2007), 137 and 379 Angus (Marques et al., 2008; de Roos et al., 2008, respectively). Additionally LD information on CH and C beef cattle is still limited in the current literature. Reported in this paper are the results for the extent of LD and persistence of LD phase in purebred AN, CH, and C beef cattle, as well as effective population sizes for the two purebreds.

## Materials and methods

### Animals

The purebred animals consisted of 597 AN and 450 CH steers born on 2004–2009, originating from the Onefour Research Substation of the Agriculture and Agri-Food Canada Research Centre at Lethbridge, presently located at the Kinsella Research Ranch, University of Alberta. The pedigrees that were made available to this study contained 1059 and 857 individuals for AN and CH, respectively. The longest ancestral path for these two populations was one. The numbers of sires for these AN and CH populations were 74 and 86, respectively. There was zero pedigree-based inbreeding among these animals. The average relatedness among individuals was estimated using the numerator relationship matrix (Dunner et al., 1998) and was approximately 0.004 for both AN and CH. AN and CH cows were bred with AN and CH sires, respectively, using artificial insemination (AI).

**Figure 1:**
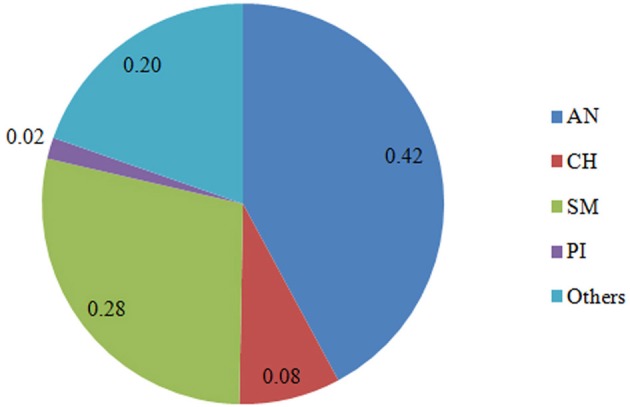
**Breed composition (%) of the crossbred animals genotyped**. AN, Angus; SM, Simental; PI, Piedmontese; CH, Charolais; Others, 15 other breeds.

Six hundred and 16 crossbred animals consisted of 384 steers born between 1998 and 2006, 218 bulls born 1995–2006, and 14 heifers born 1999–2005, at one of three University of Guelph cooperating herds: Elora Beef Research Centre, New Liskeard Agriculture Research Station, and Agriculture and Agri-Food Canada's Kapuskasing experimental Farm. Cows were bred to mostly purebred sires through the extensive use of AI. Semen (predominantly AN and Simmental [SM]) was supplied by primarily AI companies or local breeders. These test animals comprised 11 animals of 50% AN and 50% (Piedmontese) PI, 231 of 50–75% AN, 41 of 75–87.5%AN, 141 of 50–75% SM, 7 of at least 75% SM, 28 of at least 50% CH, and 157 of other breed combinations. Average breed composition of the crossbred animals genotyped for this study is presented in Figure 1. These crossbreds came from a pedigree of 4526 individuals, including 762 sires with an average of 3.17 progeny each, and 1445 dams with 2.67 progeny each. There were 113 full-sib groups with family size between 2 and 9 individuals, averaging 2.35. The longest ancestral path was 7. The average inbreeding coefficient was 0.018. The average relatedness among individuals in the pedigree was approximately 0.05.

### Genotypes

The AN and CH animals were genotyped for 54,609 single nucleotide polymorphisms (SNP), using the Illumina Bovine SNP50_v2 Beadchip; and the crossbreds were genotyped for 51,620 SNP across the bovine genome, using the Illumina Bovine SNP50_v1 Beadchip. The genotyping was accomplished on blood samples at the University of Alberta. SNPs that were out of Hardy–Weinberg equilibrium (*P* < 0.01), and/or had low call rate (<95%) were removed from further analysis. The work of Du et al. (2007) revealed that the *r*^2^ metric was slightly biased when SNP had minor allele frequency (MAF) less than 10% and came from a small sample size. Since the numbers of animals in the three populations used in this study are considered larger sample sizes, SNP with 5% MAF or above were included in analyses. After screening, 31,073, 32,088, and 33,286 SNP on 29 autosomal chromosomes were used for AN, CH, and C, respectively. SNP position from the UMD 3.1 bovine assembly was used in this study.

### LD and *N*_*e*_ estimation

One measure of LD is the difference (*D*) between the observed and the expected haplotype frequencies. *D* = *f*(*AB*) – *f*(*A*)*f*(*B*), where *f(AB)* is the estimated frequency of haplotype AB using the observed genotype frequency (McVean, 2007) and assuming Hardy–Weinberg equilibrium, while *f(A)* and *f(B)* being the frequencies of alleles *A* and *B*, respectively. However, *D* is highly dependent on allele frequencies and therefore undesirable for comparing LD among multiple pairs of loci. Hill and Roberson (1968) developed *r*^2^ as a measure of LD, $r^{2}=\frac{D^{2}}{f(A)f(a)f(B)f(b)}$, where *f(A)*, *f(a)*, *f(B)* and *f(b)* are observed frequencies of alleles *A*, *a*, *B*, and *b*, respectively. The metric *r*^2^ varies between 0 and 1, where zero means that the SNPs are completely uncorrelated while 1 means the two SNPs are perfectly correlated. The *r*^2^ metric represents the correlation of determination for alleles at 2 loci, and is proven less dependent on allele frequencies in finite population sizes as compared to other LD measures (Hedrick, 1987; Lewontin, 1988; Zapata, 2000; Abecasis et al., 2001; Mueller, 2004). It is also preferred for bi-allelic markers (Zhao et al., 2007), thus used in this study. LD phase, *r*, is the square root of *r*^2^, and bears the sign of D. Pair-wise LD (*r*^2^) was estimated on each chromosome. More details of this technique are described by Sargolzaei et al. (2008).

A linear model was set out to determine the effects of marker intervals, chromosomes, and breed groups on the decay of LD, LD_*ijk*_ = *d*_*i*_ + breed_*j*_ × BTA_*k*_ + *e*_*ijk*_, where LD_*ijk*_ was the observed LD over marker distance *d*_*i*_ for marker pair *i* of breed group *j* on chromosome *k*. The distance was fit as a covariate, using a linear model package in R (R Development Core Team, 2009). Effective population sizes for AN and CH at different periods of the population history were estimated following (Sved, 1971), $N_{e}=\left(\frac{1}{4c}\right)\;(\frac{1}{r^{2}}-1)$, where *N*_*e*_ is the effective population size, *c* the marker distance in Morgans (assuming 100,000,000 base pairs per Morgan). The age of *N*_*e*_ for any distance is estimated by $\frac{1}{2c}$ (Hayes et al., 2003).

## Results

### Effects of distance, chromosome, and breed on the decay of *r*^2^

Table 1 summarizes the SNPs analyzed in this study. The total genome length was 2,534.98–2,535.30 Mb, with the shortest *Bos taurus* autosomal chromosome (BTA) being 42.72 Mb (BTA25) and the longest 158.09Mb (BTA1). The distribution of SNPs varied among the chromosomes, with BTA1 having the highest number of SNPs (2026–2176) and BTA28 having the fewest (580–607); the longest SNP interval was identified on BTA20 (38.77 Mb). However, average SNP intervals were relatively consistent among the chromosomes, and the overall average distance between two adjacent SNPs was 70 kb.

**Table 1:** **Summary of analyzed SNP**.

**BTA**	**Length (Mb)**	**Number of SNP**	**Average SNP (Interval in Mb [SD])**	**Longest interval (Mb)**	**Shortest interval (Mb)**	***r*^2^ (SD) between adjacent SNPs**		
						**AN**	**CH**	**C**
1	158.09	2026–2176	0.07 (0.06)	0.67–0.69	2.40e-03	0.25 (0.28)	0.17 (0.22)	0.17 (0.22)
2	136.48	1677–1776	0.07 (0.07)	0.84	1.00e-06	0.23 (0.27)	0.17 (0.23)	0.16 (0.22)
3	121.09–121.14	1465–1606	0.07 (0.07)	0.96–1.03	1.00e-06	0.25 (0.27)	0.16 (0.21)	0.16 (0.21)
4	119.74–119.77	1482–1602	0.07 (0.06)	0.55	1.00e-06	0.24 (0.27)	0.16 (0.21)	0.15 (0.20)
5	121.08	1269-1381	0.08 (0.09)	1.03–2	1.48e-04	0.24 (0.27)	0.16 (0.21)	0.15 (0.20)
6	119.12–119.19	1566–1653	0.07 (0.06)	1.6–1.68	1.00e-06	0.24 (0.27)	0.18 (0.23)	0.16 (0.22)
7	112.27	1296–1415	0.07 (0.06)	1.74	1.00e-06	0.25 (0.27)	0.17 (0.23)	0.17 (0.22)
8	112.91	1488–1596	0.07 (0.06)	0.66	1.00e-06	0.24 (0.27)	0.17 (0.21)	0.16 (0.21)
9	104.94–104.99	1259–1344	0.07 (0.07)	0.67–0.74	4.49e-04–7.30e-03	0.22 (0.27)	0.16 (0.22)	0.15 (0.21)
10	103.09	1321–1392	0.07 (0.06)	3.32	1.00e-06	0.19 (0.24)	0.15 (0.21)	0.14 (0.20)
11	106.88–106.93	1320–1428	0.07 (0.06)	0.77–0.89	1.00e-06	0.24 (0.27)	0.16 (0.22)	0.16 (0.22)
12	90.92–90.94	1040–1097	0.07 (0.07)	4.48	1.00e-06	0.22 (0.25)	0.17 (0.22)	0.15 (0.21)
13	83.84	1049–1147	0.07 (0.06)	0.72-0.74	1.00e-06	0.26 (0.28)	0.15 (0.19)	0.14 (0.19)
14	83.06–83.15	1129–1212	0.07 (0.06)	1.05–1.16	1.00e-06	0.26 (0.29)	0.19 (0.24)	0.17 (0.22)
15	84.14–84.22	995–1084	0.07 (0.06)	0.77–0.85	1.00e-06	0.22 (0.26)	0.15 (0.21)	0.14 (0.20)
16	81.25	967–1059	0.07 (0.07)	1.4–1.52	1.00e-06	0.23 (0.26)	0.15 (0.21)	0.15 (0.21)
17	74.67–74.89	979–1043	0.07 (0.06)	1.18–1.23	1.00e-06	0.19 (0.24)	0.14 (0.20)	0.13 (0.19)
18	64.97–65	794–846	0.07 (0.06)	1.14–1.17	1.00e-06	0.23 (0.27)	0.15 (0.21)	0.14 (0.20)
19	63.47	832–873	0.07 (0.06)	0.71	1.01e-03	0.21 (0.24)	0.14 (0.19)	0.12 (0.18)
20	110.08	943–1020	0.07 (0.06)	38.78	1.00e-06	0.22 (0.25)	0.14 (0.19)	0.14 (0.19)
21	71.1	856–914	0.07 (0.07)	1.32–2.11	1.00e-06	0.21 (0.24)	0.15 (0.19)	0.15 (0.20)
22	61.22	762–830	0.07 (0.06)	0.47–0.68	2.43e-03	0.21 (0.26)	0.15 (0.21)	0.14 (0.20)
23	51.73–52.07	691–723	0.06 (0.05)	0.78	1.00e-06	0.19 (0.23)	0.13 (0.18)	0.13 (0.19)
24	62.1	763–795	0.07 (0.06)	0.45	1.00e-06	0.23 (0.27)	0.15 (0.21)	0.14 (0.19)
25	42.72	625–651	0.06 (0.05)	0.4	1.35e-03	0.20 (0.25)	0.14 (0.20)	0.13 (0.19)
26	50.78–50.95	638–700	0.07 (0.05)	0.39–0.4	1.00e-06–2.80e-04	0.19 (0.23)	0.13 (0.20)	0.12 (0.18)
27	45.37	589–623	0.07 (0.06)	1.47	1.00e-06	0.21 (0.25)	0.13 (0.18)	0.13 (0.19)
28	46.12	580-607	0.07 (0.06)	0.56	1.12e-02	0.21 (0.24)	0.14 (0.19)	0.14 (0.19)
29	51.1	666-693	0.07 (0.06)	1.6	1.00e-06	0.18 (0.22)	0.14 (0.19)	0.13 (0.17)

**Figure 2:**
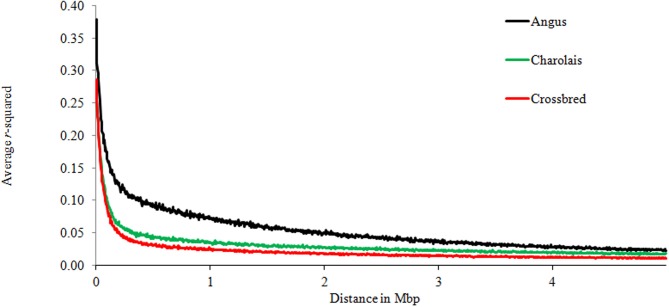
**Average LD over genomic distance for Angus, Charolais, and Crossbred**.

Table 2 shows the significant effects of genomic distances, breed groups, and chromosomes, as well as the interaction between breeds and chromosomes on the amount of LD. To display graphically the decay of LD, distances of pair-wise LD were binned into 5 kb intervals with the first bin being 10 kb large (e.g., 0–10 kb, 10–15 kb, 15–20 kb, 20–25 kb) along the first 5 Mb of each chromosome, and average *r*^2^ was computed for each interval. Figure 2 presents LD decay over varying distances of the genome. The measured LD was high for pairs of SNPs within close proximity. AN appeared to have consistently higher LD and lower rate of LD decay than CH and C at all times.

**Table 2:** **Effects of distance, breed, BTA on measured LD**.

	**Df**	**Sum sq**.	**Mean sq**.	***F*-value**	**Significance**
Breed	2	4.341	2.17	19503.86	^***^
Chromosome	28	0.763	0.027	245.038	^***^
Distance	1	25.005	25.005	224708.6	^***^
Breed × Chromosome	56	0.157	0.003	25.231	^***^
Residuals	1480305	164.723	0.000111		

* p < 0.001.

The average *r*^2^ for SNPs separated by intervals ≤30 kb were 0.29, 0.22, and 0.21 for AN, CH and C, respectively (Table 3). In that same breed group order 34.62, 26.04, and 25.87% of SNP pairs had *r*^2^ greater than 0.3. For the 30–70 kb interval, the mean *r*^2^ (percentage of pairs) with LD at least 0.3 were 0.23 (27.78%), 0.16 (7.82%), 0.15 (6.71%) for AN, CH, and C respectively. As the distance between SNPs increased, *r*^2^ decreased rapidly. The same linear model as performed earlier was used in each distance range, and showed that breed groups had significant influence on the decay of LD.

**Table 3:** **LD over varied distances**.

**Distance range (kb)**		**Number of SNP pair**	**Average *r*^2^ (SD)^§^**	**SNP pair with *r*^2^ > 0.3 (%)^§^**	**Significant effect of breed group**
0–30	AN	6608	0.29 (0.30)	2288 (34.62)	^***^
	CH	6970	0.22 (0.25)	1815 (26.04)	
	C	7498	0.21 (0.24)	1940 (25.87)	
30–70	AN	17,388	0.23 (0.26)	4831 (27.78)	^***^
	CH	18,342	0.16 (0.21)	3269 (17.82)	
	C	19,786	0.15 (0.20)	3307 (16.71)	
70–100	AN	12,832	0.19 (0.23)	2720 (21.20)	^***^
	CH	13,651	0.12 (0.17)	1519 (11.13)	
	C	14,680	0.11 (0.16)	1448 (9.86)	
100–200	AN	41,599	0.14 (0.19)	6240 (15.00)	^***^
	CH	44,271	0.08 (0.12)	2446 (5.53)	
	C	47,507	0.07 (0.11)	2098 (4.42)	
200–1000	AN	322,517	0.09 (0.12)	21981 (6.82)	^***^
	CH	343,293	0.04 (0.07)	3778 (1.10)	
	C	369,198	0.03 (0.05)	1870 (0.51)	

§ Pairwise LD;

*** p < 0.001.

The decay of LD was found to be significantly different among chromosomes as well. The rate of LD decay was slower on BTA5 and 13, but more rapid on BTA29 than the average of the whole genome. This association in AN is presented in Table 4. The average LD for distances less than 30 kb was 0.29 for the whole genome, but higher for BTA5 and 13 (0.32 and 0.34, respectively), and lower for BTA29 (0.24). As LD was averaged over more extended distances, the trend between chromosomes remained (Table 4). There were consistently more SNP pairs with LD at least 0.3 on BTA5 and 13 than the whole genome average.

**Table 4:** **LD observed in Angus on BTA5, 13, and 29**.

**Distance (kb)**	**BTA**	**Number of SNP pairs**	**Average *r*^2^ (SD)**	**SNP pair with *r*^2^ > 0.3 (%)**	**Compared to the overall mean *r*^2^**
0–30	overall	228	0.29 (0.30)	79 (34.73)	–
	5	213	0.32 (0.32)	81 (38.03)	NS
	13	206	0.34 (0.32)	87 (42.23)	^*^
	29	143	0.24 (0.23)	46 (32.17)	NS
30–70	overall	600	0.23 (0.26)	167 (27.84)	–
	5	613	0.26 (0.30)	192 (31.32)	^*^
	13	572	0.26 (0.28)	186 (32.52)	^*^
	29	399	0.19 (0.23)	81 (13.21)	NS
70–100	overall	442	0.19 (0.23)	94 (21.31)	–
	5	479	0.20 (0.24)	117 (19.09)	NS
	13	435	0.22 (0.26)	118 (27.13)	^**^
	29	280	0.14 (0.17)	39 (6.36)	NS
100–200	overall	1434	0.14 (0.19)	216 (15.06)	–
	5	1420	0.18 (0.21)	310 (50.57)	^***^
	13	1405	0.17 (0.20)	294 (20.93)	^***^
	29	898	0.10 (0.14)	82 (13.38)	NS
200–1000	overall	11,121	0.09 (0.12)	763 (6.86)	–
	5	11,458	0.12 (0.15)	1286 (11.22)	^***^
	13	10,759	0.11 (0.14)	1006 (9.35)	^***^
	29	7082	0.07 (0.09)	230 (3.25)	NS

* p < 0.05

** p < 0.01

*** p < 0.0001

NS, not significant.

**Figure 3:**
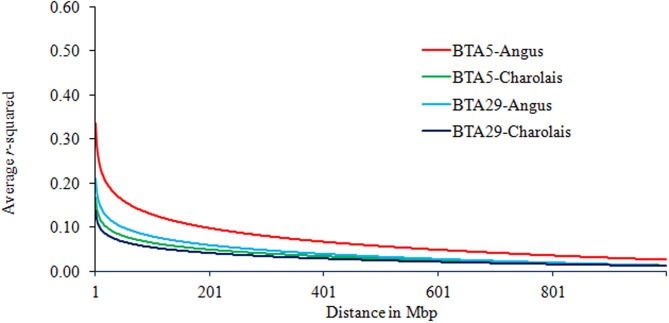
**Trend lines of average LD over genomic distance on chromosomes 5 and 29 in Angus and Charolais**.

In terms of interaction between breeds and chromosomes, the rate of LD decay was delayed the most on BTA5 as compared to other chromosomes. Yet this was observed in AN only and is presented in Figure 3. The average *r*^2^ on BTA5 of AN was higher than that of CH while the average LD on BTA29 and its decay were similar in both breeds, especially at genomic distance greater than 1 Mb.

### Phase of LD

Pearson correlation coefficient for LD phase between same pairs of SNP, for pairs of breeds was obtained and presented in Table 5. The correlation was high over short distances and decreased as the distance expanded. For all SNP pairs, the correlation between AN and CH was as high as 0.77 for distances of 70 kb or less. This number was higher between AN and C (0.84), as well as CH and C (0.81). This is expected because more than one-third of the crossbred animals in the current study were at least 50% AN. The correlation was even higher (as high as 0.97 and 0.94 between AN and CH, AN and the crossbreds, respectively, for distances of 200 kb or less) for SNP pairs with LD at least 0.3. This is important because if a QTL is found in a chromosome region in AN, markers linked to the QTL have 94% chance to carry the same effect in the crossbred animals in this population given that region has an LD of at least 0.3.

**Table 5:** **Correlation of LD phase among breed groups**.

	**0–70 kb**		**70–100 kb**		**100–200 kb**	
	**CH**	**C**	**CH**	**C**	**CH**	**C**
AN	0.77 (0.98^§^)	0.84 (0.91^§^)	0.61 (0.97^§^)	0.81 (0.94^§^)	0.45 (0.97^§^)	0.75 (0.90^§^)
CH	NA	0.81 (0.91^§^)	NA	0.72 (0.94^§^)	NA	0.59 (0.90^§^)

§ Correlation of phase for SNP pairs with LD at least 0.3.

### Effective population size

**Figure 4:**
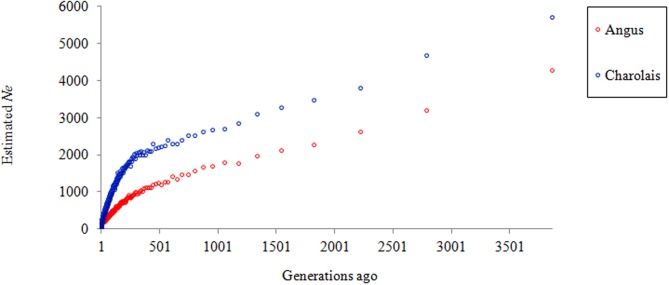
**Estimated *N*_*e*_ for Angus and Charolais over time**.

Figure 4 shows a clear trend in declining *N*_*e*_ in both AN and CH. At almost all times the effective population size of CH was higher than that of AN and the reduction rate in *N*_*e*_ was consistent until approximately 250 generations ago, when it began to accelerate and became even more rapid in the past 100 generations. This may suggest a bottleneck has occurred in both breeds, plus the use of AI in the more recent past leading to the *N*_*e*_ of approximately 207 and 285 for AN and CH, respectively, 10 generations ago. Compared to the estimates by Villa-Angulo et al. (2009), the current study estimated higher effective population size for AN (207 vs. 64 individuals), and CH (285 vs. 130 individuals) for 10 generations ago.

## Discussion

The sample sizes of AN and CH in the current study were approximately 10 times larger than those used for LD studies by McKay et al. (2007), and Watanabe et al. (2008), and 20 times larger than the AN and CH populations used in a study by Villa-Angulo et al. (2009). Beside the current study used a much larger number of markers, approximately 32,000 SNPs, creating roughly 28,000, 15,000, and 48,000 SNP pairs over distances of 0–70, 70–100, and 100–200 kb, respectively, to estimate the metric *r*^2^*.* Therefore, the haplotype samples used in this study were believed to well represent the breed populations. The average *r*^2^ for AN was lower than values reported for this breed by de Roos et al. (2008), Villa-Angulo et al. (2009), and McKay et al. (2007), even over some short distances. The same trend was observed on CH average *r*^2^ as compared to reported values by Villa-Angulo et al. (2009) and McKay et al. (2007). This could be attributed in part to the difference in sample sizes between the current study and previously reported research. To support this, the current study estimated *r*^2^ from a small group of 60 purebred AN, using the same SNP panel, and found average *r*^2^ to be consistent with the values reported for this breed by de Roos et al. (2008), Villa-Angulo et al. (2009), and McKay et al. (2007) (results not shown).

Higher LD was found for BTA5, but only in the AN population. This may reflect selection for traits that are strongly influenced by QTL on this chromosome in this breed. AN is a popular breed in Canadian beef production and genetic trends suggest strong selection for growth and other performance traits (American Angus Association, 2012). Additionally, AN are medium to small size cattle, selection for better growth could be stronger for AN than for CH, which have bigger body sizes. Various studies have shown highly significant evidences for the presence of QTLs affecting birth weight (Li et al., 2002; Kim et al., 2003) and carcass traits (Stone et al., 1999; Casas et al., 2000; Kim et al., 2003) on BTA5. The QTL for growth and carcass traits could be attributed to the insulin-like growth factor-1 gene or to one or more surrounding genes, such as the myf5 gene on BTA5 (Kim et al., 2003; Li et al., 2004; Islam et al., 2009). In addition, when selection operates at a locus, the neighboring loci in close linkage with the locus under selection will have an enhanced extent of LD. When selection occurs at multiple loci in epistasis, LD between loci under epistatic selection and their tightly linked loci will be created and enhanced (Du et al., 2007).

Useful LD is commonly understood as that of sufficiently large degree to be used in an LD mapping, or more recently in a genomic selection program. The threshold for useful LD may depend on applications and the nature and accuracy of trait phenotype measurements (Du et al., 2007). For LD mapping purposes, Du et al. (2007) suggested an *r*^2^ of at least 0.3 be sufficient in swine. In the current study, any pair of adjacent SNPs spanned an average distance of approximately 70 kb, and in AN 49% (*versus* 38% and 37% in CH and C, respectively) of SNP pairs separated by 70 kb or less showed *r*^2^ of at least 0.15. Subsequently if a QTL is located halfway between two SNPs of a pair then the LD amount between that QTL and each of the two SNP would be twice the correlation between the SNPs, and thus might be at least 0.3. If the *r*^2^ threshold of 0.3 is applicable to useful LD in beef cattle, and required for genomic selection to achieve a high accuracy for genomic BV (Meuwissen et al., 2001) suggested an LD of at least 0.2 for an accuracy of 0.85), then the current SNP density would allow the capture of approximately 49% of useful LD between SNP and QTL in AN (*versus* 38% and 37% in CH and C, respectively). This accuracy could be improved by redesigning the SNP chip based on LD blocks, or increasing marker density in regions of low LD, or regions of interest. To illustrate this, further analysis (calculation not shown) showed that the amount of useful LD between SNP and QTL in AN, CH, and C could be increased to 54 and 43%, respectively, given a panel of 84,500 usable SNPs. This improvement would be substantially enhanced with the availability of bovine half million SNP panels as SNP intervals could be narrowed down to approximately 215 b for any two adjacent SNPs. Two such panels, Illumina BovineHD BeadChip (Illumina Inc, 2010) and Axiom Genome-Wide BOS 1 Array (Affymetrix Inc., 2011) are now available.

**Figure 5:**
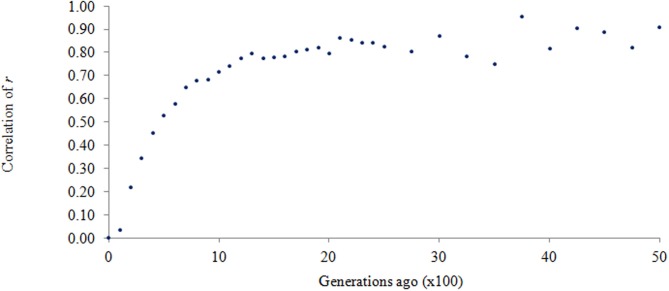
**Correlation of *r* between Angus and Charolais over past generations**.

In terms of the persistence of LD phase, the correlation of *r* represents the genetic relationship between the populations (de Roos et al., 2008). In the current study *r* was consistently high (at least 0.90) for any pairwise comparison of the three breed groups, given that LD was at least 0.3. This is interesting because if a QTL is in linkage with its surrounding SNPs, and its effect is estimated in a training population then used to predict trait performance in a different population, the direction of the effect is highly preserved if the correlation of *r* for the two populations is high as discovered in this study. Since LD over a short genomic distance is considered historical LD, the current data reveals a close genetic relationship between AN and CH over short genomic distance, and indicates these two breeds came from the same population thousands of generations ago. To illustrate this, the correlation of *r* for the two breeds was plotted over past generations in Figure 5. Very high correlation of *r* suggests that these two breeds were genetically close to each other thousands of generations ago. This is supported by a common belief that *Bos primigenius* was the last common ancestor of domestic cattle (Friend, 1978). A rapid decline in *r* approximately 1500 generations ago, as shown in Figure 5, suggests a divergence between AN and CH; then the slope of the curve becomes steeper and steeper toward the recent past. It could be that the original domestication of cattle, followed by differential breed development, led to the level of genetic separation observed today.

Hill (1972) proposed a formula for estimating *N*_*e*_ = 4NL/(2 + σ^2^_*n*_), where N is the total number of animals alive at anytime, L being the generation length, σ^2^_*n*_ the variance of family size. Using this relationship the shrinkage of *N*_*e*_ depends on the number of sires and the variance of progeny number per sire. Mukai et al. (1989) and Nomura et al. (2001) found that the latter played a larger role in the decrease of the population size. To maximize the net response in economic merit for dairy cattle, Goddard and Smith (1990) suggested a minimum effective number of 10 bull sires per generation, equivalent to 40 individuals per generation. FAO (1998) recommended an effective population size of 50 per generation to maintain the fitness in a breed. The estimated *N*_*e*_ 10 generations ago for both AN and CH in the current study were well above the recommended numbers. This could be attributed to a sufficiently large number of sires being used to produce animals in the current dataset, and thus a small variance of family size. However, the slope of the *N*_*e*_ in Figure 4 suggests that the population sizes were decreasing consistently fast, possibly due to the use of AI, and therefore actions are needed to maintain sufficiently large *N*_*e*_.

## Conclusion

The amount of LD decayed rapidly as SNP pair distance increased within 200 kb, but the LD over longer distances remained consistently low. For a given genomic distance, populations ranked as AN, CH, C animals for level of LD. The phase of LD was more persistent between AN and the C animals than between AN and CH, as well as CH and the C. This persistence was very high between any pair of the three breed groups for SNP pairs with LD as large or larger than 0.3. The behavior of the correlation of *r* indicates AN and CH came from one common population thousands of generations ago; their genetic divergence started approximately 1500 generations ago and accelerated over the past 250 generations. The estimated *N*_*e*_ for AN and CH 10 generations ago were 207 and 285, respectively, and sufficiently large to maintain fitness and maximize responses to selection for economic traits. The study also reveals a redesign of the current SNP chip or an increase in SNP density is necessary to exploit more useful LD for genome-wide selection in a population consisting of these breeds.

### Conflict of interest statement

The authors declare that the research was conducted in the absence of any commercial or financial relationships that could be construed as a potential conflict of interest.
